# An Examination of Iron-Deficiency Anemia and Its Associated Risk Factors Among Libyan Workers and Their Families

**DOI:** 10.7759/cureus.78606

**Published:** 2025-02-06

**Authors:** Asma A Alboueishi, Fawzi O Ebrahim, Saber Dalyom, Mohamed Breem, Warda Haroush, Sundus Alshileeb, Faihaa Omran, Adam Elzagheid

**Affiliations:** 1 Department of Cell Biology and Cell Culture, Libyan Biotechnology Research Center, Tripoli, LBY; 2 Department of Genetic Engineering, Libyan Biotechnology Research Center, Tripoli, LBY

**Keywords:** iron-deficiency anemia, serum ferritin, serum iron, total iron-binding capacity, workers

## Abstract

Introduction: Iron deficiency is primarily the cause of anemia, a major public health problem worldwide. This study aimed to determine the prevalence of iron-deficiency anemia (IDA) and its risk factors in working men and women.

Methods: This cross-sectional study was conducted between June and August 2023. The study included employees of the Biotechnology Research Center in Tripoli, Libya, and their family members. The sample consisted of 67 male and 134 female participants aged 12-74 years. Each participant completed a questionnaire for sociodemographic data. Two blood samples were collected from each participant to analyze the iron profile and complete blood count. All the participants signed an informed consent form before enrollment. IDA was defined according to World Health Organization criteria. Lifestyle items that might be associated with iron deficiency were also investigated. Descriptive statistics were calculated, and a comparison of groups was made with an independent t-test, risk ratios, and 95% confidence intervals (CIs).

Results: The mean age ± standard deviation of the study sample was 38.35 ± 13.97 years; 33% of the participants were men. The overall prevalence of IDA was 34% (95% CI, 32.3-35.6). Moreover, IDA prevalence was higher in women (44.78%; 95% CI, 43.1-45.3) than in men (13.43%; 95% CI, 12.2-14.0) (p* *< 0.001). There was a corresponding significant difference between the male and female participants in serum ferritin, serum iron, and total iron-binding capacity (p* *< 0.001). Age, marital status, residence, occupational status, dietary habits, and family history of IDA were the main risk factors (p < 0.001).

Conclusion: The prevalence of IDA was moderate and substantial among men (13.43%). It was particularly high among working women (44.78%). There were significant associations between IDA and sociodemographic and lifestyle factors, including age, marital status, residence, occupational status, dietary habits, and family history of IDA.

## Introduction

Iron-deficiency anemia (IDA) is the most common micronutrient deficiency in the developing world, resulting from a long-term negative iron balance. It is particularly more common among children, women of childbearing age, and individuals with chronic diseases. The World Health Organization (WHO) has stated that approximately two billion people globally are affected by anemia, with 50% of all cases being IDA [1].

The prevalence of iron deficiency in the Middle East is similar to that observed in other developing countries, with estimates ranging from 25% to 35%. In contrast, the prevalence in developed countries falls between 5% and 8% [2]. IDA can be caused by inadequate iron intake, poor iron absorption, blood loss, and some chronic medical conditions [3].

The reference range for normal blood hemoglobin levels varies depending on sex, age, and geographical area. Children, elderly people, pregnant and lactating women, and people with diseases causing blood loss are at a higher risk than other groups of the population [4]. The prevalence of IDA is also related to cultural habits, socioeconomic factors, and developmental characteristics. IDA adversely affects physical activity and work performance [5]. This study aimed to determine the prevalence and risk factors associated with IDA among men and women employees of the Biotechnology Research Center in Tripoli, Libya, and their family members.

## Materials and methods

This cross-sectional observational study was performed among employees of the Biotechnology Research Center in Tripoli, Libya, and their family members of all ages between June and August 2023. Excluded were persons with incomplete information, children, pregnant or breastfeeding women, and persons taking medication or nutritional supplements. Ethical approval was obtained from the Committee of Research Ethics at the Biotechnology Research Center (ref no. BEC-BTRC 8-2023). The participants were informed in advance of the study objectives and protocol. They provided written informed consent and were assured of the privacy of the results. A questionnaire was completed by each participant, along with their family members, to collect sociodemographic data (age, marital status, place of residence, occupational status, and family income), as well as nutritional habits and smoking. History of IDA and chronic diseases were recorded.

Blood samples were collected for analysis. Anemia was defined according to WHO criteria: a hemoglobin concentration of <12 g/dL in nonpregnant women and <13 g/dL in men. Furthermore, the severity of anemia was classified as mild (Hgb, 11-11.9 g/dL in women and 11-12.9 g/dL in men), moderate (Hgb 8-10.9 g/dL) and severe (Hgb <8 g/dL) [6]. A Beckman Coulter LH 520 Analyzer (Beckman Coulter, Inc., Brea, CA) was used to determine the complete blood count, and Roche Cobas E411 (Roche Diagnostics, Mannheim, Germany) was used to measure serum ferritin (SF), serum iron (SI), and total iron-binding capacity (TIBC). IDA was defined as SF <15 ng/mL for women and <25 ng/mL for men, SI <50 µg/mL in women and <65 µg/mL in men, and TIBC ≥250 µg/dL for men and women alike.

The data were analyzed using Statistical Package for the Social Sciences version 26.0 (IBM Corp., Chicago, IL). Results are presented as mean, standard deviations, and median. The prevalence of IDA is reported as a percentage, risk ratio (RR), and 95% CI. A comparison of groups was done with an independent t-test for hematological parameters. Chi-square test and CIs for each variable were then generated to assess the association of each risk factor with the participants’ anemia status. A difference was considered statistically significant for a p value of <0.001 or <0.005.

## Results

Sixty-seven men and 134 women aged 12-74 years participated in the study. The mean age of men was 43.87 ± 13.67 years, and that of women was 35.59 ± 13.33 years. Fifty-eight men (86.57%) were nonanemic with normal average hemoglobin (14.79 ± 1.11 g/dL), while only nine men were anemic (hemoglobin <13.0 g/dL). IDA was highly prevalent in women, with 44.78% of them (95% CI, 43.1-45.3) having hemoglobin values <12.0 g/dL. Similarly, the mean of SF, SI, and TIBC for IDA men and women participants had a low significant level compared to nonanemic men and women participants groups (p < 0.001) (Table 1).

**Table 1:** Levels of Hgb, MCV, SF, SI, and TIBC parameters and prevalence of IDA according to age and sex Data are presented as mean ± standard deviation, median (interquartile range), or n (%) p values were calculated using independent t-test IDA: iron-deficiency anemia; RBC: red blood cell count; Hgb: hemoglobin; HCT: hematocrit; MCV: mean corpuscular volume; MCH: mean corpuscular hemoglobin; MCHC: mean corpuscular hemoglobin concentration; RDW: red cell distribution width; SF: serum ferritin; SI: serum iron; TIBC: total iron-binding capacity

Parameters	Non-IDA (n = 132)		IDA (n = 69)		p value
	Male	Female	Male	Female	
Gender	58 (86.6)	74 (55.2)	9 (13.4)	60 (44.8)	0.001
RBC (×10¹²/L)	5.21 (5.19)	4.71 (4.66)	4.48 (4.57)	4.24 (4.22)	0.001
Hgb (g/dL)	14.79 ± 1.11	13.05 ± 1.03	11.32 ± 1.09	10.02 ± 1.10	0.001
HCT (%)	45.81 (45)	40.03 (40)	36.97 (37)	34.07 (34.6)	0.001
MCV (fL)	88.30 (88)	84.92 (86)	78.55 (75)	72.34 (73)	0.001
MCH (pg)	29.75 (30)	28.22 (29)	25.92 (26)	24.30 (25)	0.001
MCHC	33.45 (34)	32.90 (33.05)	31.09 (31)	29.81 (30)	0.001
RDW (%)	14.20 (13.8)	13.68 (14)	15.40 (13.19)	15.63 (16)	0.001
Serum ferritin (ng/mL)	95.22 (68.8)	41.47 (27.50)	39.56 (12)	10.43 (10)	0.001
Serum iron (ug/mL)	104.8 (102.5)	89.20 (82)	51.67 (51)	23.36 (28.5)	0.001
Total binding iron capacity (ug/mL)	178.4 (188.5)	184.96 (203)	255.8 (256)	265.9 (266.5)	0.001

**Figure 1:**
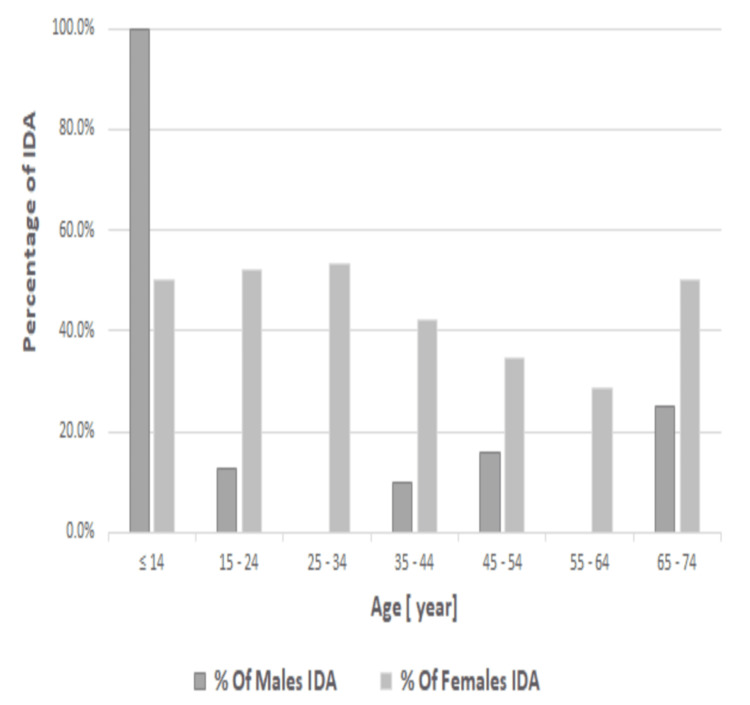
Prevalence of IDA in male and female participants according to age IDA: iron-deficiency anemia

The IDA prevalence was remarkably higher among women than men in all age groups. The prevalence of IDA was decreasing with the increase in women aged <14-64 years; nonetheless, it rises in the elderly aged 65-75 years. In men, it increased gradually with increasing age and was most prominent among individuals aged 65-74 years (25%) (Figure 1).

**Figure 2:**
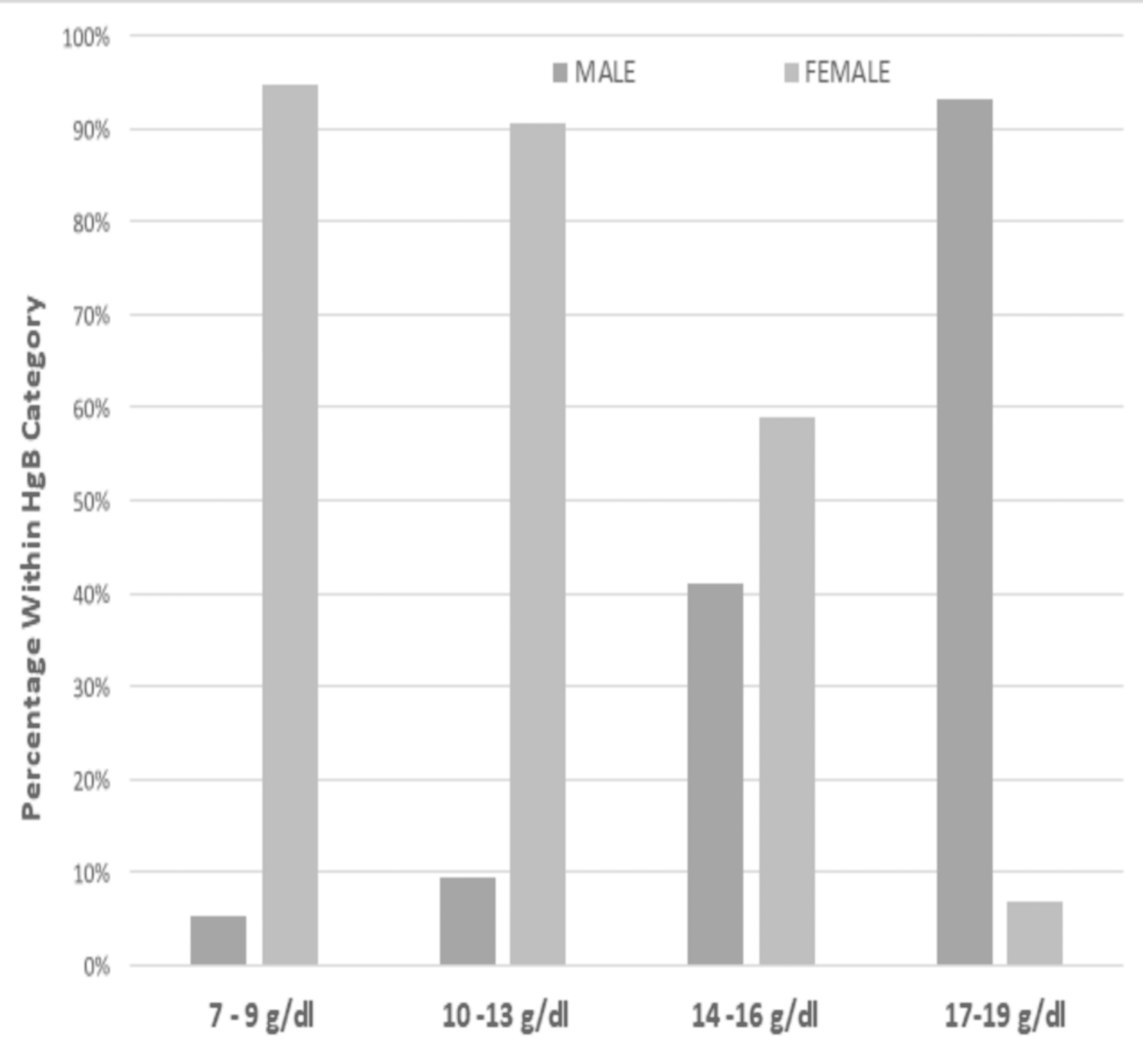
Distribution of hemoglobin concentrations in all participants Hgb: hemoglobin

Male participants had a much higher prevalence of higher hemoglobin levels, indicating a better hemoglobin status than female participants. Most male participants (93%) had hemoglobin levels in the range of 17-19 g/dL, and 41% had normal hemoglobin levels in the range of 14-16 g/dL. In addition, a smaller percentage (9%) had hemoglobin levels in the range of 10-13 g/dL, indicating mild anemia. A small percentage of male participants (5%) had low hemoglobin levels in the range of 7-9 g/dL, indicating moderate-to-severe anemia. However, a significantly higher percentage of female participants than male participants fell into the lower Hgb categories (7%-9% and 10%-13%); a smaller percentage (7%) had high hemoglobin in the range of 17-19 g/dL (Figure 2).

Regarding anemia severity, the most common degrees of anemia were moderate (49.3%, n = 34) and mild (47.8%, n = 33). Severe anemia was rare, with only two participants (2.9%) having Hgb <8.0 g/dL.

The associations between IDA and various parameters are shown in Table 2. Those who were married were more likely to have IDA (p < 0.001) but less likely in women who were divorced (p < 0.005). The prevalence of IDA in participants living in rural areas was more likely to develop IDA than those in urban areas (odds ratio, OR, 1.46; p < 0.001). Employed participants were more likely to have IDA (p < 0.001). Underweight individuals were more likely to have IDA than normal-weight and overweight individuals (p < 0.001). In addition, the prevalence of IDA was higher in men and women who consumed tea, coffee, or cola frequently (p < 0.001). It should be noted that 15.0% of men and 53.3% of women mentioned having a family history of IDA (p < 0.001). There were no associations of IDA with the other variables.

**Table 2:** Distribution of participants with IDA and nonanemic participants according to sex and potential risk factors The RRs and 95% CIs were generated from the cross-tabulation test. Significance level was at p < 0.01 or p < 0.05 IDA: iron-deficiency anemia; BMI: body mass index; RR: risk ratio; CI: confidence interval; Ref: reference

Variables	Male participants			Female participants			p value
	Normal	IDA	RR (95% CI)	Normal	IDA	RR (95% CI)	
Sociodemographics							
Age							
≤14	0 (0)	1 (100)	2.75 (2.6-3.34)	3 (2.2)	3 (50)	0.50 (0.92-2.07)	<0.001
15-24	7 (10.4)	1 (12.5)	Ref	11 (8.2)	12 (52.2)	1.46 (1.28-1.65)	<0.001
25-34	3 (4.5)	0 (0)	Ref	15 (11.2)	17 (53.1)	1.72 (1.41-1.74)	<0.001
35-44	18 (26.9)	2 (10)	2.68 (2.37-2.99)	22 (16.4)	16 (42.1)	1.47 (1.25-1.69)	<0.001
45-54	21 (31.3)	4 (16)	2.80 (2.51-2.89)	17 (12.7)	9 (34.6)	0.65 (0.45-1.84)	<0.001
55-64	6 (9)	0 (0)	Ref	5 (3.7)	2 (28.6)	0.71 (0.26-2.16)	<0.001
65-74	3 (4.5)	1 (25)	2.50 (0.90-4.09)	1 (0.7)	1 (50)	0.850 (0.50-7.85)	<0.001
Marital status							
Single	14 (87.5)	2 (12.5)	092 (0.25-3.39)	38 (57.6)	28 (42.4)	0.57 (0.45-1.69)	-
Married	44 (86.3)	7 (13.7)	1.02 (0.70-1.49)	35 (54.7)	29 (45.3)	1.54 (1.42-1.67)	<0.001
Divorced	0	0	Ref	1 (25)	3 (75)	1.25 (0.45-2.04)	<0.002
Widowed	0	0	Ref	0	0	Ref	-
Residence							
Urban	29 (93.5)	2 (6.5)	0.44 (0.12-1.55)	42 (65.6)	22 (34.4)	0.64 (0.43-0.95)	-
Rural	29 (80.6)	7 (19.4)	1.55 (1.00-2.40)	32 (45.7)	38 (54.3)	1.46 (1.49-1.71)	<0.001
Occupational status							
Working	51 (87.9)	7 (12.1)	2.76 (2.59-2.93)	46 (60.5)	30 (39.5)	1.60 (1.49-1.71)	<0.001
Unemployed	1 (100)	0 (0)	Ref	12 (36.4)	21 (63.6)	1.36 (1.19-1.53)	<0.001
Student	5 (71.4)	2 (28.6)	1.43 (1.53-2.33)	15 (62.5)	9 (37.5)	0.62 (0.41-1.83)	-
Retired	1 (100)	0 (0)	Ref	1 (100)	0 (0)	Ref	-
BMI							
Underweight	6 (75)	2 (25)	1.45 (0.17-1.55)	10 (40)	15 (60.1)	1.32 (0.97-1.33)	<0.001
Normal	29 (90.6)	3 (9.4)	Ref	37 (58.7)	26 (41.3)	Ref	-
Overweight	18 (85.7)	3 (14.3)	Ref	22 (61.1)	14 (38.9)	Ref	-
Obese	5 (83.3)	1 (16.7)	Ref	5 (50)	5 (50)	Ref	-
Smoking							
Yes	17 (94.4)	1 (5.6)	1.25 (0.97-1.61)	0 (0)	1 (100)	0.25 (0.94-1.67)	0.078
No	41 (83.7)	8 (16.3)	0.37 (0.05-2.44)	74 (55.6)	59 (44.4)	0.98 (0.95-1.01)	-
Family income							
Very good	21 (87.5)	3 (12.5)	0.80 (0.51-1.09)	22 (73.3)	8 (26.7)	2.88 (2.61-3.14)	0.317
Good	34 (85)	6 (15)	1.93 (1.86-2.01)	51 (52)	47 (48)	2.91 (2.81-3.01)	-
Low	3 (1)	0 (0)	Ref	1 (16.7)	5 (83.3)	2.89 (2.78-3.01)	0.080
Breakfast							
Regular	30 (78.9)	8 (21.1)	1.71 (1.22-2.41)	33 (47.1)	37 (52.9)	1.99 (0.99-3.99)	<0.074
Irregular	28 (96.6)	1 (3.4)	0.23 (0.03-1.48)	41 (64.1)	23 (35.9)	0.69 (0.47-1.01)	0.036
Tea consumption							
≥3×/day	34 (79.1)	9 (20.9)	Ref	44 (49.4)	45 (50.6)	0.61 (0.36-1.03)	<0.001
≤2×/day	24 (100)	0 (0)	1.70 (1.37-2.11)	30 (66.7)	15 (33.3)	1.26 (0.99-1.60)	-
Cola consumption							
Yes	30 (81.1)	7 (18.9)	1.50 (0.97-2.30)	43 (49.4)	44 (50.6)	1.26 (1.98-1.61)	0.001
No	28 (93.3)	2 (6.7)	0.46 (0.13-1.60)	31 (66)	16 (34)	0.63 (0.38-1.04)	-
Coffee consumption							
Yes	47 (90.4)	5 (9.6)	2.34 (1.94-2.38)	47 (53.4)	41 (46.6)	1.07 (0.84-1.37)	<0.001
No	11 (73.3)	4 (26.7)	0.68 (0.37-1.24)	27 (58.7)	19 (41.3)	0.86 (0.53-1.40)	-
Physical activity							
Yes	24 (100)	0 (0)	0.70 (1.37-2.11)	14 (60.9)	9 (39.1)	1.04 (0.91-1.19)	<0.132
No	34 (79.1)	9 (20.9)	0.20 (0.19-1.11)	60 (54.1)	51 (45.9)	0.79 (0.39-1.57)	<0.163
Family history of IDA							
Yes	7 (100)	9 (15)	1.13 (1.03-1.25)	14 (46.7)	16 (53.3)	1.41 (0.75-2.65)	<0.001
No	51 (85)	0 (0)	Ref	60 (57.7)	44 (42.3)	0.90 (0.74-1.09)	-
Family history of chronic diseases							
Yes	20 (90.9)	2 (9.1)	1.18 (0.79-1.76)	13 (76.5)	4 (23.5)	1.13 (0.99-1.28)	<0.085
No	38 (84.4)	7 (15.6)	0.64 (0.18-2.30)	61 (52.1)	46 (47.9)	0.37 (0.13-1.10)	-

## Discussion

Prior studies on anemia in Libya have focused mainly on women of childbearing age and children [7,8]. Our study is the first to examine the prevalence and risk factors for IDA in working men and women in Libya. The overall prevalence of IDA was 34%, and the most common grade was moderate anemia (49.3%). The WHO estimates that up to 27% of the world's population has IDA [9]. In our study, the prevalence of IDA was higher among working women (44.78%) than working men (13.43%), which agrees with a recent study by Shah et al., in which a significant association between anemia and employment in women (41.7% were anemic) was noted [10]. Additionally, previous studies [11,12] have highlighted similar trends, suggesting that the higher prevalence rates in women may be due to iron loss during menstruation or poor nutritional status. The higher prevalence rates in women may be due to iron loss during menstruation or poor nutritional status. Our study also found significant positive associations between IDA status and various parameters. Being married was a risk factor for IDA (p < 0.001), which was more prevalent in women than in men (45.3% vs. 13.7%). The lower iron level in women could be due to their increased demand for iron supplies during pregnancy and lactation [13,14].

The high prevalence of IDA among rural men and women compared to urban dwellers (19.4% vs. 6.5% in men and 54.3% vs. 34.4% in women; p < 0.001) was similar to that reported in Ethiopia, which described a 46.6% prevalence in rural residents and 20.1% in urban residents [4].

Normal and overweight participants were less likely to have IDA, whereas underweight women were 60.1% more likely to have IDA (OR, 1.32; p < 0.001) [15,16]. This was not reported in Mexican women [17]. Likewise, we found no association between body mass index and anemia in men.

Age was significantly associated with IDA. Overall, the IDA prevalence was remarkably higher among women than men in all age groups [15]. In women, we observed that the prevalence of IDA gradually decreased with increasing age, particularly in childbearing age [18,19]. These findings are consistent with other studies conducted in the childbearing group of women in Saudi Arabia. This finding was further corroborated by a previous study of men. There is a steadily increasing trend in the prevalence of IDA with age. In Canada, the rate of IDA rises as age increases, peaking at 30% among men over 50 years old [20,21], attributed to aging, inflammation, and chronic diseases.

Tea, coffee, and cocoa contain polyphenols (tannins) that inhibit iron absorption from the intestine, which may contribute to developing IDA [22]. Phenolic compounds can form complexes with iron in the intestine, making it unavailable for absorption. Nearly all beverages reduced iron absorption, with black tea being the most potent, reducing iron absorption by 79%-94% [23]. In our study, there were strong statistically significant associations between tea and coffee consumption and iron absorption (p < 0.001) [24,25]. Similarly, cola consumption significantly increased the risk of IDA (p < 0.001) [26].

Moreover, a family history of IDA was significantly associated with increased odds of IDA, especially in female participants (p < 0.001); those with a family history of IDA likely had an increased chance of having IDA [27].

Our study has certain limitations, particularly its cross-sectional design and small sample size. These factors may impact the generalizability of our findings, as a limited sample may not fully represent the broader population.

## Conclusions

The prevalence of IDA was moderate among men (13.43%). It was particularly high among working women (44.78%). There were significant associations between IDA and sociodemographic and lifestyle factors, including age, marital status, residence, occupational status, dietary habits, and family history of IDA. Additional studies should be carried out annually, particularly on working women and men, to assess the prevalence of IDA and the causative factors for higher prevalence, whether due to lack of awareness of anemia, work-related factors, or malnutrition. The inclusion of different age groups and a larger number of participants from different regions, both working and unemployed, would be beneficial for a clearer understanding. Frequent studies will provide the opportunity for intervention and education to reduce the incidence of anemia in men and women who are working.
